# Spermidine promotes retinal ganglion cell survival and optic nerve regeneration in adult mice following optic nerve injury

**DOI:** 10.1038/cddis.2015.93

**Published:** 2015-04-16

**Authors:** T Noro, K Namekata, A Kimura, X Guo, Y Azuchi, C Harada, T Nakano, H Tsuneoka, T Harada

**Affiliations:** 1 Visual Research Project, Tokyo Metropolitan Institute of Medical Science, Tokyo, Japan; 2 Department of Ophthalmology, The Jikei University School of Medicine, Tokyo, Japan

## Abstract

Spermidine acts as an endogenous free radical scavenger and inhibits the action of reactive oxygen species. In this study, we examined the effects of spermidine on retinal ganglion cell (RGC) death in a mouse model of optic nerve injury (ONI). Daily ingestion of spermidine reduced RGC death following ONI and sequential *in vivo* retinal imaging revealed that spermidine effectively prevented retinal degeneration. Apoptosis signal-regulating kinase-1 (ASK1) is an evolutionarily conserved mitogen-activated protein kinase kinase kinase and has an important role in ONI-induced RGC apoptosis. We demonstrated that spermidine suppresses ONI-induced activation of the ASK1-p38 mitogen-activated protein kinase pathway. Moreover, production of chemokines important for microglia recruitment was decreased with spermidine treatment and, consequently, accumulation of retinal microglia is reduced. In addition, the ONI-induced expression of inducible nitric oxide synthase in the retina was inhibited with spermidine treatment, particularly in microglia. Furthermore, daily spermidine intake enhanced optic nerve regeneration *in vivo.* Our findings indicate that spermidine stimulates neuroprotection as well as neuroregeneration, and may be useful for treatment of various neurodegenerative diseases including glaucoma.

Traumatic optic neuropathy is a common clinical problem that occurs in 0.5–5% of patients with closed head injury.^1^ A damage to the optic nerve causes shear stress and induces secondary swelling within the optic canal, accompanied by subsequent RGC loss and optic nerve atrophy.^2^ Although no large natural history or randomized controlled trial has been published, neither corticosteroid therapy nor optic canal decompression surgery is considered as standard treatments for patients with traumatic optic neuropathy,^3^ and there is a lack of effective treatment at present. Research into finding therapeutic targets for treatment of traumatic optic neuropathy indicated that neuroprotection and axon regeneration may be effective strategies and studies using an optic nerve injury (ONI) model in rodents have provided useful information. For example, neurotrophins, such as brain-derived neurotrophic factor and ciliary neurotrophic factor, protect retinal ganglion cells (RGCs) and promote axon regeneration in an ONI model.^4, 5, 6^ In addition, inhibition of neuroinflammatory events such as upregulation of tumor necrosis factor (TNF)-*α* and nitric oxide synthase (NOS) may be effective for RGC protection following ONI.^7^ The ONI model mimics some aspects of glaucoma, including RGC death induced by excitotoxicity and oxidative stress, and therefore it is also a useful animal model for glaucoma.

Glaucoma is one of the leading causes of vision loss in the world and it is estimated that this condition will affect more than 80 million individuals worldwide by 2020, with at least 6–8 million individuals becoming bilaterally blind.^8^ Glaucoma is characterized by progressive degeneration of RGCs and their axons, which are usually associated with elevated intraocular pressure, but there is a subset of glaucoma termed normal tension glaucoma (NTG) that presents with statistically normal intraocular pressure. There are several animal models of glaucoma, including DBA/2J mice,^9^ and inducible models such as cauterization of episcleral veins.^10, 11, 12^ In addition, we previously reported that loss of glutamate transporters (EAAC1 or GLAST) in mice leads to RGC degeneration that is similar to NTG^13^ and these animal models have been useful in examining potential therapeutic targets.^14, 15, 16^

Spermidine is naturally and almost exclusively accumulated in glial cells in the brain and retina.^17, 18^ It acts as an endogenous free radical scavenger and inhibits the action of reactive oxygen species. Indeed, it has been reported that spermidine has key roles in mediating protection against oxidative damage caused by hydrogen peroxide in cultured mouse fibroblasts^19^ and administration of spermidine extended the lifespan of yeast, flies, worms and human immune cells by upregulating the lysosomal/vacuolar degradation pathway, referred to as autophagy, which leads to enhanced resistance to oxidative stress and decreased cell death.^20^ Previously, we reported that oral administration of spermidine ameliorates severity of experimental autoimmune encephalomyelitis, an animal model of multiple sclerosis, by suppression of oxidative stress,^21^ suggesting that spermidine may also be effective in protecting RGCs from increased oxidative stress associated with various pathogenic conditions in the eye including traumatic optic neuropathy and glaucoma.

In this study, we examined the effects of daily spermidine intake on ONI-induced retinal degeneration. We monitored changes in retinal morphology over a course of 2 weeks following ONI, using optical coherence tomography (OCT), which permits noninvasive, longitudinal and quantitative assessment of retinal structures in living animals. We also explored possible mechanisms associated with spermidine-mediated neuroprotection.

## Results

### Spermidine prevents RGC death following ONI

**Figure 1:**
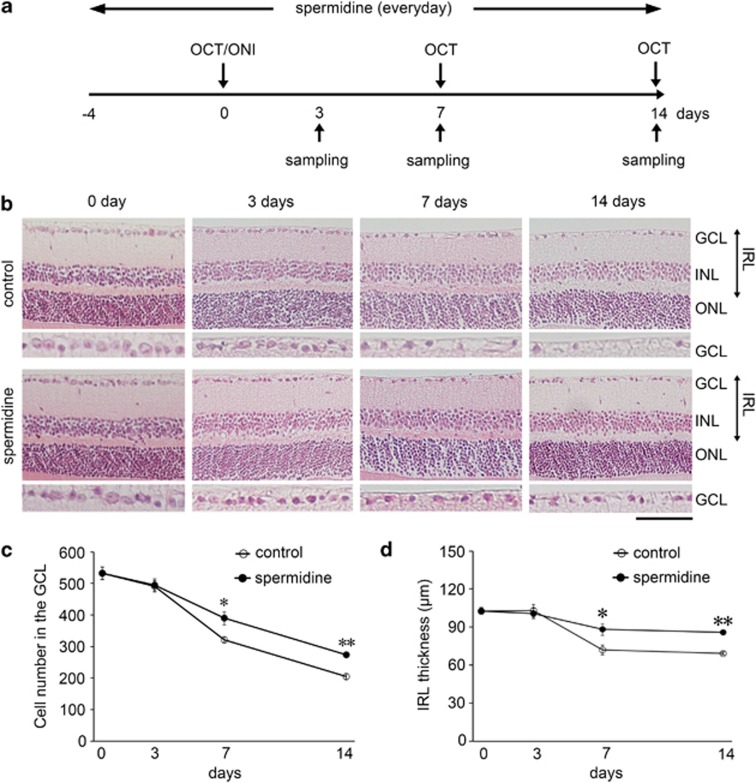
Effects of spermidine on RGC loss and IRL thickness following ONI. **(a)** Experimental protocols. Spermidine (30 mM) was added to drinking water for the treatment group from 4 days before ONI throughout the experimental period and the control group received normal drinking water. The treatment period was completed on d14 after ONI. **(b)** Retinal sections stained with hematoxylin and eosin at d3, 7 and 14 after ONI in control and spermidine-treated mice. Scale bar: 100 and 50 *μ*m in the upper and immediately lower panels, respectively. GCL, ganglion cell layer; INL, inner nuclear layer; IRL, inner retinal layer; ONL, outer nuclear layer. Quantification of the RGC number **(c)** and IRL thickness **(d)** in control and spermidine-treated mice. The data are presented as means±S.E.M. of six samples for each experiment. *P<0.05, **P<0.01

We first investigated neuroprotective effects of spermidine in the retina following ONI. Spermidine (30 mM) was given to adult mice in their drinking water for 18 days starting from 4 days before ONI through to 14 days after ONI (Figure 1a). By day 7 (d7) and d14 following ONI, the number of RGCs was significantly reduced, but spermidine treatment suppressed such RGC loss (Figures 1b and c). In addition, the thickness of the inner retinal layer (IRL) was significantly decreased in vehicle-treated mice following ONI, whereas this effect was prevented in spermidine-treated mice (Figure 1d). These results suggest that spermidine protects RGCs from ONI-induced cell death.

### Spermidine suppresses retinal degeneration following ONI

**Figure 2:**
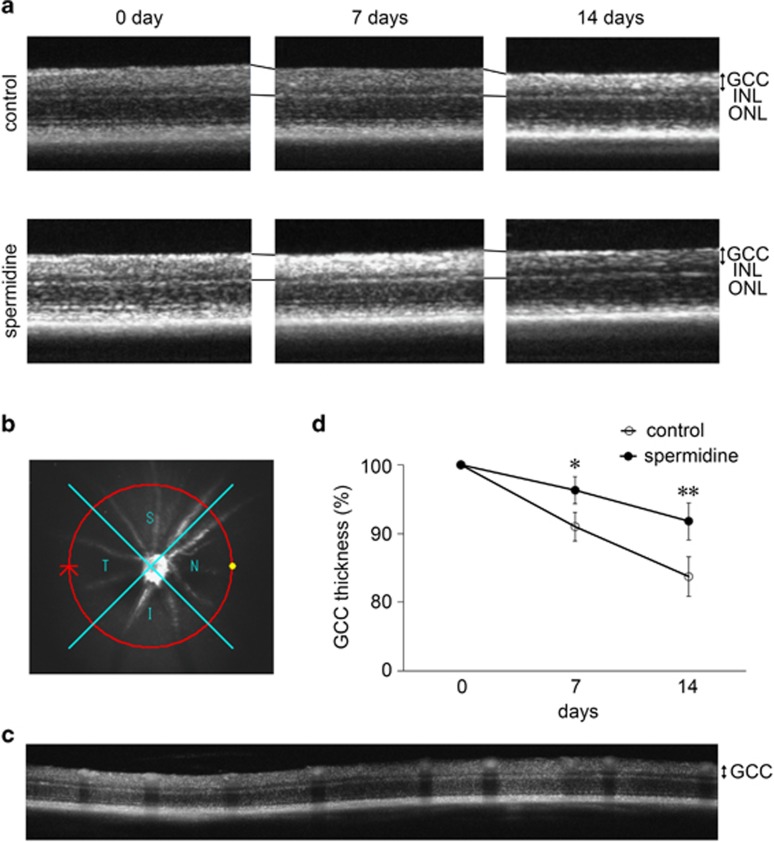
*In vivo* imaging of the retina in control and spermidine-treated mice. **(a)** OCT cross-sectional images of retinas at d0, 7 and 14 following ONI. GCC, ganglion cell complex. **(b)** An image of a circle centering around the optic nerve disc. **(c)** An OCT circular scan image captured from **b**. **(d)** Longitudinal evaluation of the GCC thickness by a circular scan. The data are presented as means±S.E.M. of six samples for each experiment. *P<0.05, **P<0.01

We also visualized retinal layers using OCT, a non-invasive imaging technique that can be used to acquire cross-sectional tomographic images of the retina *in vivo*.^22^ This technique is very useful in monitoring the changes in retinal structures over a period of time following injury in living animals.^15, 16, 22, 23, 24^ The average thickness of the ganglion cell complex (GCC), which includes the nerve fiber layer, ganglion cell layer (GCL) and inner plexiform layer, was decreased in vehicle-treated mice over the course of 2 weeks following ONI, and this effect was milder in spermidine-treated mice (Figure 2a). For quantitative analyses, GCC was measured by scanning the retina in a circle, centering around the optic nerve disc (Figure 2b), and the average GCC thickness was determined from acquired images (Figure 2c). The quantitative analyses confirmed that the reduction in the GCC thickness following ONI is significantly suppressed with spermidine treatment (Figure 2d). These results suggest that spermidine suppresses ONI-induced retinal degeneration.

### Spermidine inhibits activation of the ASK1-p38 pathway in RGCs following ONI

**Figure 3:**
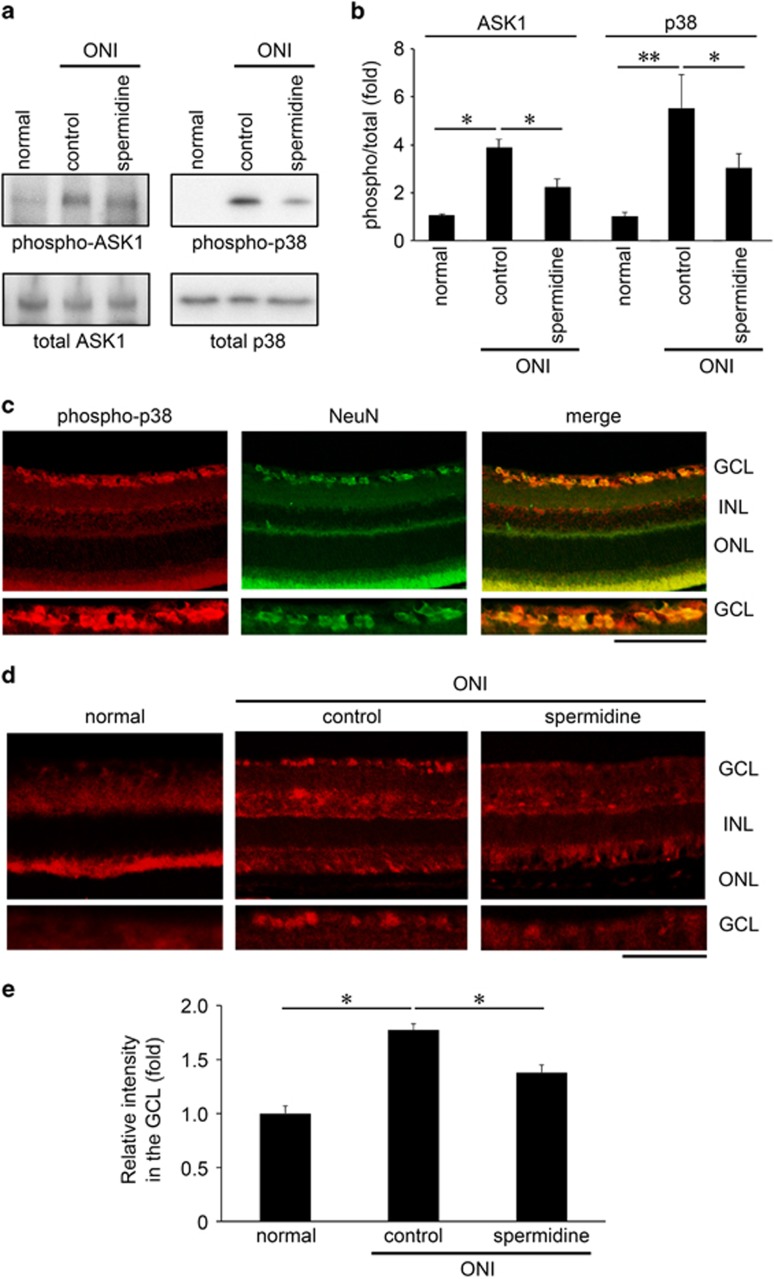
Effects of spermidine on ONI-induced activation of the ASK1 signaling pathway in the retina. **(a)** Effects of spermidine on activation of the ASK1-p38 pathway in whole retinas at 4 h after ONI. **(b)** Quantitative analyses of ONI-induced activation of ASK1 and p38 in whole retinas in **a**. **(c)** Immunohistochemical analyses of ONI-induced p38 phosphorylation (red) and NeuN expression (green) in the retina. Overlapping immunoreactivities (yellow) indicate p38 phosphorylation in RGCs. Scale bar: 200 and 100 *μ*m in the upper and immediately lower panels, respectively. **(d)** Immunohistochemical analyses of ONI-induced retinal p38 phosphorylation with or without spermidine treatment. Scale bar: 200 and 100 *μ*m in the upper and immediately lower panels, respectively. **(e)** Quantitative analyses of **d**. Data are normalized to the phospho-p38 intensity at the GCL in normal mice. The data are presented as means±S.E.M. of six samples for each experiment. *P<0.05, **P<0.01

We recently reported that the apoptosis signal-regulating kinase-1 (ASK1)-p38 pathway is involved in ONI-induced RGC death.^22^ ASK1 has key roles in human diseases closely related to dysfunction of cellular responses to oxidative stress and endoplasmic reticulum stressors, including neurodegenerative diseases.^14, 25^ As spermidine possesses anti-oxidant properties, we examined whether spermidine modulates the ASK1-p38 pathway. Following ONI, the retinal expression of phosphorylated (phospho)-ASK1 and p38 were significantly increased, but spermidine inhibited these changes (Figures 3a and b). Immunohistochemical analyses revealed that the increase in phospho-p38 expression following ONI was detected mainly in the GCL, which was doubled-labeled with NeuN (a RGC marker) (Figure 3c). Spermidine treatment clearly suppressed this increased expression levels of phospho-p38 (Figures 3d and e). These results suggest that spermidine inhibits ONI-induced activation of the ASK1-p38 pathway in RGCs, which leads to RGC protection.

### Spermidine decreases chemokine productions and microglial accumulation following ONI

**Figure 4:**
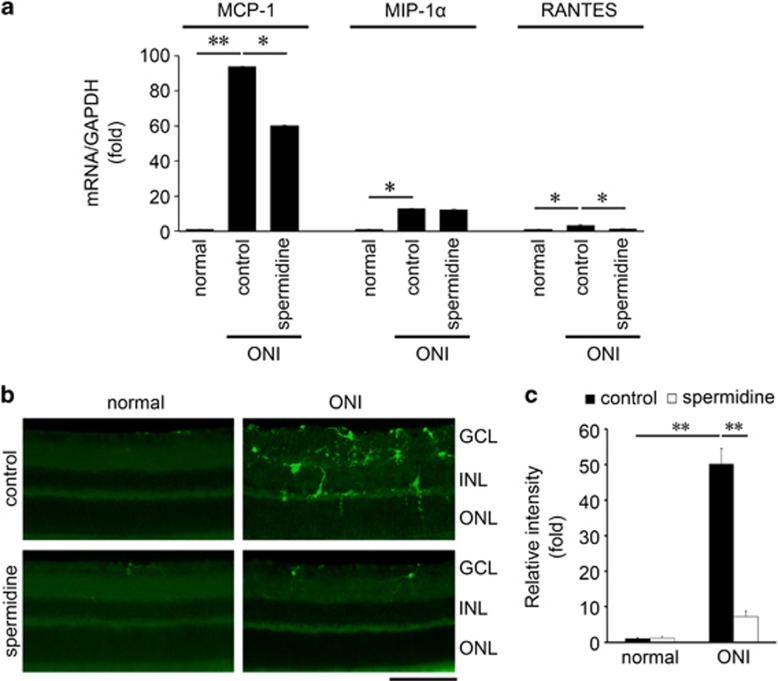
Effects of spermidine on ONI-induced upregulation of chemokines and accumulation of microglia. **(a)** mRNA expression levels of MCP-1, MIP-1α and RANTES in whole retinas at d4 after ONI were determined using quantitative real-time PCR. Glyceraldehyde 3-phosphate dehydrogenase (GAPDH) was used as an internal control. **(b)** Immunohistochemical analyses of ONI-induced migration of microglia detected with an iba1 antibody in the retina at 0 h and d7 after ONI. Scale bar: 100 *μ*m. **(c)** Quantitative analyses of **b**. Data are normalized to the iba1 intensity in normal mice. The data are presented as means±S.E.M. of six samples for each experiment. *P<0.05, **P<0.01

We explored other potential mechanisms associated with spermidine-mediated neuroprotection. We previously reported that ONI increases retinal expressions of various chemokines implicated in the pathogenesis of neuroinflammation.^22^ Therefore, we examined the ONI-induced changes in monocyte chemoattractant protein-1 (MCP-1), macrophage inflammatory protein 1*α* (MIP-1*α*) and regulated on activation, normal T-cell-expressed and -secreted (RANTES) expression levels in the retina. As expected, the mRNA levels of MCP-1, MIP-1*α* and RANTES were all increased in the retina following ONI. We found that the expressions of examined chemokines were also elevated in the spermidine-treated mouse retina, but the extent of increase was significantly less for MCP-1 and RANTES (Figure 4a). Spermidine did not affect the retinal MIP-1*α* expression level. As MCP-1 and RANTES are involved in migration and activation of microglia, we examined the presence of microglia in the retina following ONI with or without spermidine treatment. Immunohistochemical analyses revealed that ONI induced a significant increase in the number of microglia in the retina at d7 and spermidine treatment almost completely inhibited this effect (Figures 4b and c). These results suggest that spermidine suppresses production of chemokines important for activation and migration of microglia, resulting in RGC protection in the ONI model.

### Spermidine suppresses the inducible NOS expression in microglia following ONI

**Figure 5:**
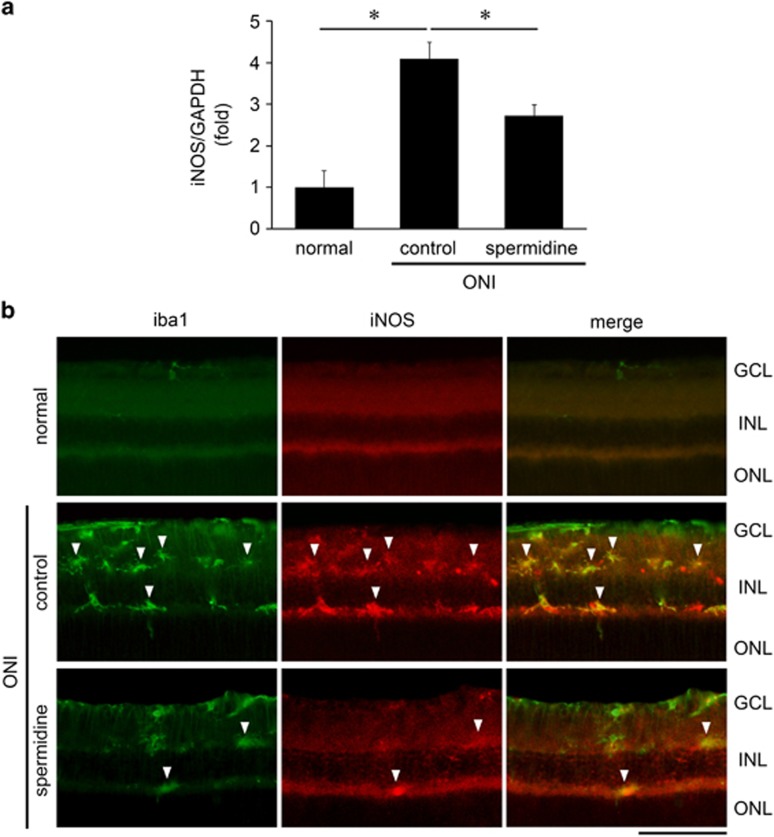
Effects of spermidine on ONI-induced iNOS activation in the retina. **(a)** mRNA expression levels of iNOS at d5 after ONI were determined using quantitative real-time PCR. Glyceraldehyde 3-phosphate dehydrogenase (GAPDH) was used as an internal control. The data are presented as means±S.E.M. of six samples for each experiment. *P<0.05. **(b)** Double-labeling immunohistochemistry of iba1 (green) and iNOS (red) at d5 after ONI. Overlapping immunoreactivities (yellow, arrowheads) indicate the activated microglial cells that produce iNOS. Scale bar: 100 *μ*m

We next investigated whether spermidine affects any cellular events within microglia. As the inducible NOS (iNOS) expression level is upregulated in activated microglia, which can cause RGC death,^26, 27^ we examined whether spermidine modulates the level of microglial iNOS expression following ONI. We first found that ONI induced a significant increase in the iNOS expression level in the whole retina, which was suppressed with spermidine treatment (Figure 5a). Immunohistochemical analyses revealed that ONI increased the number of immunopositive (IP) cells for both iba1 and iNOS in the inner retina and the extent of increase was less in the spermidine-treated retina (Figure 5b). The iNOS-IP cells were double labeled with iba1, suggesting that iNOS is produced in microglia in response to ONI, which is detrimental to RGC survival, and spermidine exerts neuroprotective effects by reducing the number of activated microglia and suppressing iNOS upregulation.

### Spermidine enhances optic nerve regeneration

**Figure 6:**
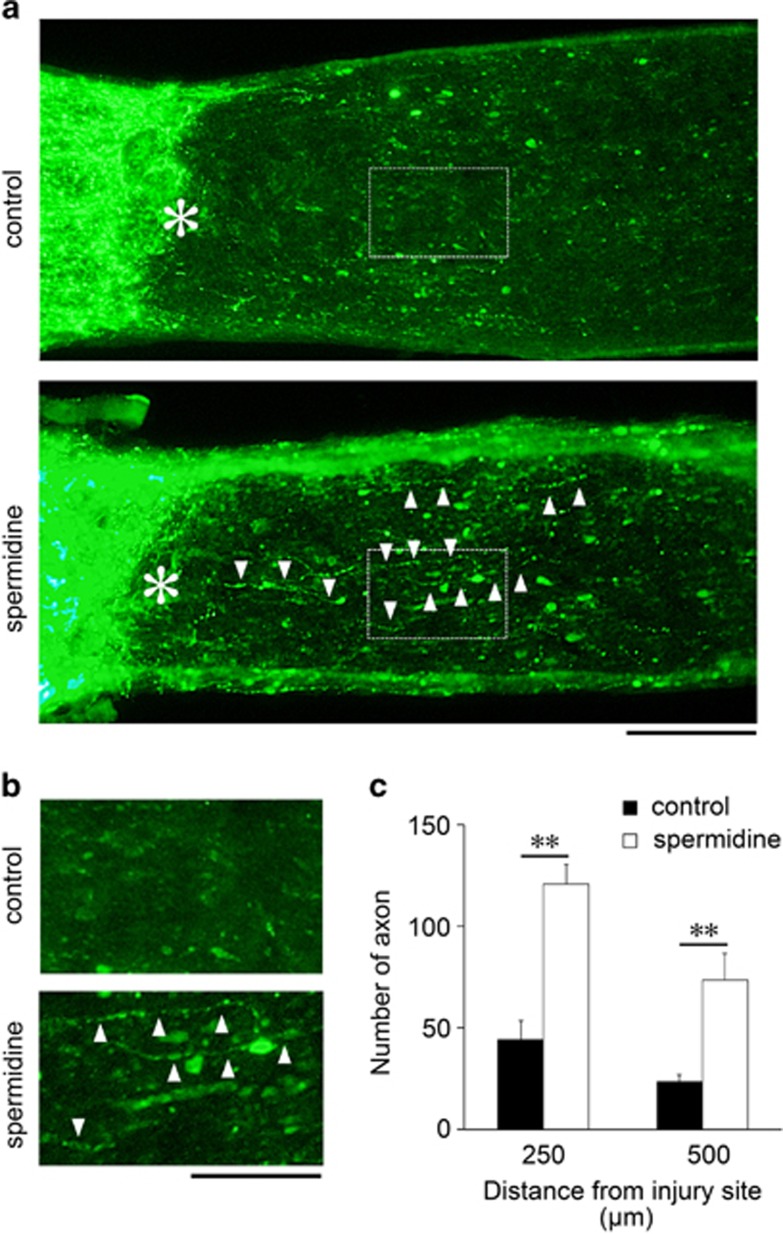
Effects of spermidine on optic nerve regeneration following ONI *in vivo*. **(a)** Longitudinal sections through the optic nerve showing CTB-positive axons distal to the injury site (*) in control and spermidine-treated mice at d14 after ONI. Arrowheads indicate regenerating axons. Scale bar: 100 *μ*m. **(b)** Higher magnification of the images shown in **a**. Scale bar, 50 *μ*m. **(c)** Quantitative analyses of regenerating axons extending 250 and 500 *μ*m from the injury site at d14 after ONI. The data are presented as means±S.E.M. of six samples for each experiment. **P<0.01

As spermidine promotes RGC survival following ONI, we hypothesized that it may have beneficial effects on optic nerve regeneration. Optic nerve regeneration is an important area of research for development of treatment strategies for conditions that damage the optic nerve, as well as the retina, including glaucoma. The regenerating axons of RGCs were traced by injecting cholera toxin *β*-subunit (CTB) conjugated to Alexa 488 into the vitreous of the retina at d11 after ONI (Figure 6a). Following ONI at d14, the number of regenerating axons in spermidine-treated mice was much greater than the control mice (Figures 6a and b). The quantitative analyses demonstrated that the number of regenerating axons that extended beyond 250 *μ*m was approximately 45 in control compared with about 120 with spermidine treatment, and half of the axons in each conditions extended beyond 500 *μ*m. These results suggest that spermidine enhances the number of regenerating axons, probably due to the increased RGC survival rate, but it may have a limited effect on the regenerative ability of axons.

## Discussion

In this study, we reported that oral administration of spermidine exerts neuroprotective effects in an ONI model. We demonstrated that daily ingestion of spermidine protects RGCs from ONI-induced cell death and retinal degeneration. We also showed that spermidine suppresses ONI-induced activation of the ASK1-p38 pathway in RGCs, which is one of the potential mechanisms involved in spermidine-mediated neuroprotection. In addition, we showed that production of chemokines important for recruitment of microglia is decreased with spermidine treatment and, consequently, accumulation of retinal microglia is reduced. ONI-induced iNOS expression in the retina was inhibited with spermidine treatment, in particular in microglia. Furthermore, daily spermidine intake enhanced optic nerve regeneration *in vivo*, suggesting that spermidine may stimulate neuroprotection as well as neuroregeneration, and may be useful for treatment of various conditions including traumatic optic neuropathy and glaucoma.

The ASK1-p38 pathway is activated in response to multiple types of stress, such as oxidative stress and endoplasmic reticulum stress.^28^ It is implicated in diseases including cancer, Alzheimer's disease and multiple sclerosis.^29^ It also has a role in RGC death and optic nerve degeneration induced under various conditions.^14, 22, 30, 31^ Considering the antioxidant properties of spermidine, it is plausible that spermidine suppressed ONI-induced activation of the ASK1-p38 pathway in RGCs, because it reduces the oxidative stress level.^19, 20, 21^ Oxidative stress is an important risk factor in human glaucoma^32^ and an antioxidant, *α*-lipoic acid, delivered through diet protects RGCs in DBA/2 J mice, an animal model that recapitulates the slow, progressive nature of human glaucoma.^33^ It would be interesting to investigate whether spermidine intake is also beneficial in animal models of glaucoma, including NTG.^13^

Microglia are resident macrophages in the central nervous system and retina, and are activated after injury. Activated microglia produce cytotoxic substances, such as TNF-*α*, reactive oxidative species, nitric oxide, proteases and excitatory amino acids that may induce neuronal cell death. These processes are implicated in various neurodegenerative diseases including glaucoma and Alzheimer's disease.^34, 35^ We previously demonstrated that MCP-1 is upregulated in RGCs following ONI,^22^ and in this study we showed that spermidine reduces ONI-induced upregulation of MCP-1 and RANTES, another chemotactic molecule for microglia, resulting in inhibition of microglia accumulation. As these chemokines recruit and activate microglia that result in neuronal apoptosis and exacerbation of neurodegeneration,^36, 37^ we suggest that spermidine exerts neuroprotective effects via suppression of microglia recruitment and possibly activation. However, we cannot exclude the possibility that microglial invasion to the inner retina is also associated with molecules that are released when RGCs die, rather than just MCP-1 and RANTES, being released by living RGCs. Although we cannot discard the possibility that the role of spermidine under these conditions is to reduce the number of migrating microglia and so it may not have a direct effect on cellular events within microglia, we suggest that suppression of iNOS upregulation is one of the mechanisms for spermidine-mediated neuroprotection. Taking together, it is speculated that endogenous spermidine accumulated in retinal glia may be an initial resource for RGC survival, and if such resources are depleted due to aging and trauma then exogenous spermidine may protect RGCs.^38^ Spermidine is known to act on the neural *N*-methyl-D-aspartate receptor in a bi-phasic concentration-dependent manner: at submillimolar doses it blocks the receptors, but at submicromolar levels it activates the receptors.^39^ In the current study, the concentration of spermidine used was 30 mM, which is the same dose that protected RGCs in a mouse model of multiple sclerosis.^21^ Further studies will be required to measure the concentration of extracellular spermidine near RGCs, to ensure the best outcome from spermidine treatment.

It has been shown that glial cells can not synthesize spermidine, but exogenous spermidine from blood vessels can be transported to glia by organic cation transporters and vesicular polyamine transporters.^40, 41, 42^ Therefore, spermidine supplement and the effect of polyamine treatment may depend on the status of the glial cell transport systems. Furthermore, spermidine has an important role in neural cell survival and resistance against trauma by openning the connexine gap junctions between astrocytes and making the glial syncytium functional to propagate vital molecules and ions from one glial cell to another.^43, 44^ As glia–glia and glia–neuron interactions have important roles in neuroprotection, it would be interesting to investigate whether spermidine mediates its neuroprotective effects also through stimulation or inhibition of various molecules associated with cell survival or cell death within microglia and Müller glia.^45, 46, 47^

Stimulation of optic nerve regeneration is an important area of research for restoring visual function after optic nerve damage. To date, studies have identified some key molecules that can enhance optic nerve regeneration and, strikingly, partial recovery of visual function following ONI has been achieved.^48, 49, 50^ We show that neuroregeneration is enhanced with daily spermidine intake *in vivo*, which is consistent with previous findings demonstrating spermidine-mediated stimulation of neurite outgrowth *in vitro* and *in vivo*.^51^ The significance of our current findings is that we administered spermidine by oral ingestion *ad libitum*, which is therapeutically more viable than by intravitreal injection that was used in the previous study. It is important to note that spermidine increased the number of regenerating axons, but the increase in length was limited. These results suggest that the effects of spermidine on axon regeneration depend on increasing the RGC survival rate, rather than enhancing the ability of axons to grow.

Spermidine is a natural component of our diet and several foods are known to contain high levels of spermidine, including soy beans, tea leaves and mushrooms. Evidence shows that eating food that is rich in spermidine results in increased blood spermidine levels,^52^ suggesting that beneficial effects of spermidine can be easily attained by making a conscious choice of food. A recent study has shown that putrescine, a precursor of spermidine, can be converted into *γ*-aminobutyric acid that makes the inhibitory pathway stronger and protects neurons from death,^53^ establishing a previously unknown neuroprotective role of polyamines in the developing brain. In conclusion, our findings indicate that spermidine intake exerts neuroprotective and neuroregenerative effects in the retina following injury, suggesting that polyamines including spermidine may be good therapeutic candidates for retinal and optic nerve degeneration, such as traumatic optic neuropathy and glaucoma.

## Materials and Methods

### Mice

Experiments were performed using 8-week-old C57BL/6J mice (CLEA Japan, Tokyo, Japan) in accordance with the Tokyo Metropolitan Institute of Medical Science Guidelines for the care and use of animals.

### Drug administration

Spermidine (Sigma, St Louis, MO, USA) was added to drinking water at 30 mM for the treatment groups throughout the whole experimental period.^21^ The control group received normal drinking water. Drinking water was replaced every 2–3 days and spermidine was freshly added from 1 M aqueous stock (spermidine/HCl, pH 7.4), which was kept at –20 °C for no longer than 1 month.

### ONI and anterograde labeling

Mice were anesthetized with sodium pentobarbital before ONI. Optic nerves were exposed intraorbitally and crushed at about 0.5–1.0 mm from the posterior pole of the eyeball, with fine surgical forceps for 5 s.^6, 22^ On d11 after ONI, 2 *μ*g of Alexa 488-conjugated CTB (Invitrogen, Carlsbad, CA, USA) was injected into the vitreous body. On d14 after ONI, the animals were perfused with Zamboni's Fixative (2% paraformaldehyde and 15% picric acid in 0.1 M phosphate buffer). The optic nerve was removed, postfixed and immersed in 30% sucrose overnight at 4 °C. The optic nerve was then embedded in an OCT compound (Sakura, Tokyo, Japan), frozen on dry ice and 10-*μ*m serial cross-sections were prepared using a cryostat and collected on MAS-coated glass slides (Matsunami, Osaka, Japan). To estimate the total number of regenerating axons, axonal outgrowth was quantified by counting CTB-positive axons that crossed a virtual line parallel to the lesion site at 250 and 500 *μ*m distal to the lesion site.^23^

### Histological and morphometric studies

Paraffin-embedded retinal sections of 7 *μ*m thickness were cut through the optic nerve and stained with hematoxylin and eosin. The RGC number and the extent of retinal degeneration were quantified in two ways.^54^ First, the number of neurons in the GCL was counted from one ora serrata through the optic nerve to the other ora serrate. Second, in the same sections the thickness of the IRL (between the internal limiting membrane and the interface of the outer plexiform layer and the outer nuclear layer) was analyzed.

### Imaging acquisition of spectral-domain OCT

Spectral-domain OCT (SD-OCT; RS-3000, Nidek, Aichi, Japan) examinations were performed at d0, 7 and 14 after ONI, with modifications.^15, 22^ For fundus imaging, polymethyl methacrylate contact lenses optimal for mice (UNICON, Osaka, Japan) were placed on the corneas. Use of the contact lenses prevents anesthesia-induced cataract progression. A 60-D adaptor lens was placed on the objective lens of the Multiline OCT to focus on the mouse retina. For imaging of the IRLs on SD-OCT using the speckle noise-reduction method, line scans and a circular scan around the optic disc were performed. All the line scan images were location matched, scanning vertically through the center of the optic nerve head at three disc diameter lengths above the optic nerve head. All the circular scan images were obtained by scanning a circle centering around the optic nerve disc. The average thickness of GCC (between the internal limiting membrane and the interface of the inner plexiform layer and the inner nuclear layer) was measured. In this study, the maximum number of B-scans set by the manufacturer (50 for line scans and 10 for circular scans) was used for averaging.

### Immunohistochemistry

Retinas and optic nerves were examined by immunostaining as reported previously.^6, 47, 55^ Immunohistochemistry was performed using the following primary antibodies: phospho-p38 (1 : 500; V1211, Promega, Madison, WI, USA), NeuN (1 : 1000, MAB377, Millipore, Billerica, MA, USA), iba1 (1 : 700; ab5076, Abcam, Cambridge, MA, USA)^22^ and iNOS (1 : 100; 610328, BD Biosciences, San Jose, CA, USA). Quantitative analysis of the IP cell number or stained region was carried out using BZ-H1C (Keyence Software, Osaka, Japan).

### Immunoblot analysis

Immunoblotting was performed as reported previously.^22^ Membranes were incubated with an antibody against ASK1 (1 : 200; Cell Signaling Technology, Beverly, MA, USA), phospho-ASK1 (1 : 200; Cell Signaling), p38 (1 : 1000; BD Biosciences) or phospho-p38 (1 : 1000; BD Biosciences).

### Quantitative real-time PCR

Quantitative RT-PCR was performed using the ABI 7500 fast RT-PCR system (Applied Biosystems, Foster City, CA, USA) with SYBR Green PCR Master Mix (Applied Biosystems) as reported previously.^56^ Complementary DNA reverse transcribed from total RNA was amplified by using primers specific for MCP-1 (sense: 5′-AACTGCATCTGCCCTAAGGT-3′, antisense: 5′-ACGGGTCAACTTCACATTCA-3′), MIP-1*α* (sense: 5′-AGATTCCACGCCAATTCATC-3′, antisense: 5′-CAGATCTGCCGGTTTCTCTT-3′), RANTES (sense: 5′-GCCCACGTCA AGGAGTATTT-3′, antisense: 5′-TGACAAACACGACTGCAAGA-3′), iNOS (sense: 5′-ACTGTGTGCCTGGAGGTTCT-3′, antisense: 5′-GGCAGCCTCTTGTCTTTGAC-3′) and glyceraldehyde 3-phosphate dehydrogenase (sense: 5′-TGCACCACCAACTGCTTAG-3′, antisense: 5′-GGATGCAGGGATGATGTTC-3′).

### Statistics

For statistical comparison of two samples, we used a two-tailed Student's *t*-test. Data are presented as means±S.E.M. *P*<0.05 was regarded as statistically significant.

## Abbreviations

ASK1: apoptosis signal-regulating kinase 1
CTB: cholera toxin *β*-subunit
GCC: ganglion cell complex
GCL: ganglion cell layer
IP: immunopositive
INL: inner nuclear layer
iNOS: inducible nitric oxide synthase
IRL: inner retinal layer
MCP-1: monocyte chemoattractant protein-1
MIP-1*α*: macrophage inflammatory protein 1*α*
NOS: nitric oxide synthase
NTG: normal tension glaucoma
OCT: optical coherence tomography
ONI: optic nerve injury
ONL: outer nuclear layer
RANTES: regulated on activation normal T-cell expressed and secreted
RGC: retinal ganglion cell
TNF-*α*: tumor necrosis factor-*α*
